# Elevated Genetic Diversity in an F_2:6_ Population of Quinoa (*Chenopodium quinoa*) Developed through an Inter-ecotype Cross

**DOI:** 10.3389/fpls.2016.01222

**Published:** 2016-08-17

**Authors:** Ouafae Benlhabib, Noura Boujartani, Peter J. Maughan, Sven E. Jacobsen, Eric N. Jellen

**Affiliations:** ^1^Departement de Production, Protection et Biotechnologies Vegetales, Agronomic and Veterinary Institute Hassan IIRabat, Morocco; ^2^Department of Plant and Wildlife Sciences, Brigham Young UniversityProvo, UT, USA; ^3^Department of Plant and Environmental Sciences, Faculty of Life Sciences, University of CopenhagenCopenhagen, Denmark

**Keywords:** quinoa, *Chenopodium*, downy mildew resistance, breeding, genetic diversity, *Peronospora variabilis*

## Abstract

Quinoa (*Chenopodium quinoa*) is a seed crop of the Andean highlands and Araucanian coastal regions of South America that has recently expanded in use and production beyond its native range. This is largely due to its superb nutritional value, consisting of protein that is rich in essential amino acids along with vitamins and minerals. Quinoa also presents a remarkable degree of tolerance to saline conditions, drought, and frost. The present study involved 72 F_2:6_ recombinant-inbred lines and parents developed through hybridization between highland (0654) and coastal (NL-6) germplasm groups. The purpose was to characterize the quinoa germplasm developed, to assess the discriminating potential of 21 agro-morpho-phenological traits, and to evaluate the extent of genetic variability recovered through selfing. A vast amount of genetic variation was detected among the 72 lines evaluated for quantitative and qualitative traits. Impressive transgressive segregation was measured for seed yield (22.42 g/plant), while plant height and maturity had higher heritabilities (73 and 89%, respectively). Other notable characters segregating in the population included panicle and stem color, panicle form, and resistance to downy mildew. In the Principal Component analysis, the first axis explained 74% of the total variation and was correlated to plant height, panicle size, stem diameter, biomass, mildew reaction, maturation, and seed yield; those traits are relevant discriminatory characters. Yield correlated positively with panicle length and biomass. Unweighted Pair Group Method with Arithmetic Mean-based cluster analysis identified three groups: one consisting of late, mildew-resistant, high-yielding lines; one having semi-late lines with intermediate yield and mildew susceptibility; and a third cluster consisting of early to semi-late accessions with low yield and mildew susceptibility. This study highlighted the extended diversity regenerated among the 72 accessions and helped to identify potentially adapted quinoa genotypes for production in the Moroccan coastal environment.

## Introduction

Quinoa (*Chenopodium quinoa*) is an ancient Andean seed crop of exceptional nutritional, and in particular protein, quality (Koziol, 1992). Its cultivation has expanded within the past decade beyond its traditional range to more than 70 countries (Food Agriculture Organization of United Nations [FAO], 2012), although the Andean nations of Peru and Bolivia are still primary quinoa producers. Due to quinoa’s exceptional nutritional value, its capacity for adaptation to diverse agro-ecological conditions and its high commercial value, quinoa is expected to play an essential role in the UN-FAO strategy to sustainably feed the world’s growing population (Jacobsen et al., 2013).

Biologically, *C. quinoa* belongs to an allotetraploid (2*n* = 4*x* = 36) complex found throughout the New World and whose root is the North American weed pitseed goosefoot (*C. berlandieri*). At least two other independent domestication events are presumed to have given rise to Mesoamerican vegetable and seed domesticates (*C. berlandieri* ssp. *nuttaliae*; Wilson and Heiser, 1979) as well as an extinct seed crop of the ancient cultures of eastern North America, *C. berlandieri* ssp. *jonesianum* (Smith and Yarnell, 2009; Kistler and Shapiro, 2011). After dispersing to the southeastern plains of South America, the weedy taxon of that region, avian goosefoot (*C. hircinum*), is presumed to have been brought into cultivation in the region of Lake Titicaca as *C. quinoa* and, at a later time, dispersed long-range to the Araucanian region on the Pacific slope in what is now Chile (Wilson, 1990; Bruno and Whitehead, 2003). Molecular genetic analyses have confirmed that the lowland Chilean coastal material is highly diverse (Fuentes et al., 2009), yet represents a branch that is separate from the even more variable quinoa germplasm of the High Andes (Christensen et al., 2007). The coastal Chilean germplasm includes genotypes that are highly resistant to heat and are day length insensitive – critical characters for quinoa breeders and agronomists seeking to expand quinoa’s production into lowland subtropical and warm-season temperate environments around the world (Jacobsen et al., 2003).

The current work is part of a quinoa introduction and selection program initiated in 2000 in Morocco and involving Brigham Young University, the Institut Agronomique et Veterinaire Hassan II, and Copenhagen University of Denmark. Germplasm adapted to Moroccan production environments was to be identified in this long-term program through traditional breeding methodologies based upon the initial creation of highly diverse quinoa populations composed of the following: (1) in the first phase, introduction of internationally available cultivated quinoa varieties, landraces, and breeding lines; followed by, (2) introduction of breeding populations combining adaptive cultivated characteristics of lowland Chilean-origin × agronomically superior Andean Highland types; and then, (3) introduction of early-generation populations derived from disease-, pest-, and heat-resistant wild lowland × cultivated parent crosses. The primary objectives of this study, falling under phase 2 of the aforementioned project, were to (1) describe agro-morpho-phenological traits an F_2:6_ population of recombinant-inbred lines (RIL) developed from an NL-6 (female, lowland) × 0654 (male, highland) cross; (2) estimate the extent of genetic diversity present by the F_6_ generation in this population; and (3) estimate the discriminating potential and heritability of several of the evaluated traits.

## Materials and Methods

### Plant Material

The quinoa population in this study was developed at BYU, Provo (USA), through a cross between the Dutch variety NL-6 (female) and Peruvian highland line 0654 (male) initially made by Alejandro Bonifacio, and was previously described in Maughan et al. (2012) as ‘Pop39’. The NL-6 parent, which is derived from lowland Chilean germplasm, has yellow panicles, short stature, is early maturing, and is sufficiently heat-tolerant to allow for its cultivation at low elevations. Line 0654 is a highland Peruvian valley ecotype with red-purple panicles, late maturity, tall stature, and is heat susceptible. The population included in the current field evaluation consisted of 70 F_2:6_ lines plus the two parents.

### Field Characterization and Statistical Analyses

Characterization of Pop39 was performed on an experimental plot at the IAV Hassan II in Rabat, Morocco, near the laboratory to facilitate daily measurements of growth and phenology. The trial was sown indoors on January 29, transplanted to sandy loam soil in the field February 26–27, maintained through manual weeding, irrigated regularly as needed, and treated twice against aphids. No fungal control against mildew was applied. To protect against avian seed predation, an anti-sparrow net was used to cover the plots. The quinoa population was planted in two completely randomized blocks, with about 20 plants and five measurements per accession per block. Elementary plots were two rows of one-meter long and spaced 35 cm apart.

Sixteen quantitative and seven qualitative traits related to plant morphology, phenology and agronomic performance were measured. Quantitative measurements included seed diameter; plant height (at 60 days = PH60, 75 days = PH75, 90 days = PH90, and at maturity = PHM); panicle or inflorescence length (IL) and width (IW); sensitivity to downy mildew (SM); number of days to maturity (DM), and phenological stages and yield (GY) components, all of which were measured during the growing season and/or at harvest. Additional biomass data logged included main stem diameter (SD); root length (RL); aboveground fresh (FWA) and dry (DWA) weights; root fresh (FWR) and dry (DWR) weights; and overall biomass (BM). Qualitative traits used in the evaluation includes color of the seeds (ranged into a 1–10 scale by using classes of Photoshop color numerical codes), leaves (green/dark green) and stem (green/purple), and the panicle color (yellow/pink/orange/red/purple) and shape (globular versus amaranthiform).

The evaluation of downy mildew (*Peronospora variabilis*) resistance (Choi et al., 2010) was performed in the laboratory on young leaves according to the inoculation method of Mhada et al. (2015). Pathogen development was evaluated every 48 h on three leaves per accession using a 0–5 notation scale where score 0 corresponds to no lesion, 1 to small and disperse lesions with less than 1 mm diameter and no sporulation on the lower side of the leaf, 2 to clearly individualized lesions increasing in number and size with a diameter between 0.5 and 1 cm without showing any sporulation on the lower side of the leaf, 3 to brown lesions, covering less than 50% of the leaf surface with a beginning of the sporulation at the lower side, 4 to lesions of larger size, covering more than 50% of the leaf area and 5 to lesions covering more than 91% of the leaf area, with a high sporulation rating on the lower and upper area.

Collected data were subjected to descriptive analyses, analysis of variance (ANCOVA) with two factors, the accessions as a fixed factor and the blocks as a random factor, and means comparisons using the Fisher test with XLSTAT software (XLSTAT-Pro 7.5). PAST software (v. 2.16, Hammer et al., 2001) was used to perform multivariate analyses to subtract the principal components which account for much of the variance, to compute the correlations matrix and to set up the dendrogram that gathers the F_2:6_ accessions into clusters using the Unweighted Pair Group Method with Arithmetic Mean (UPGMA) procedure (Sokal and Michener, 1958).

## Results

### Qualitative Traits

**FIGURE 1:**
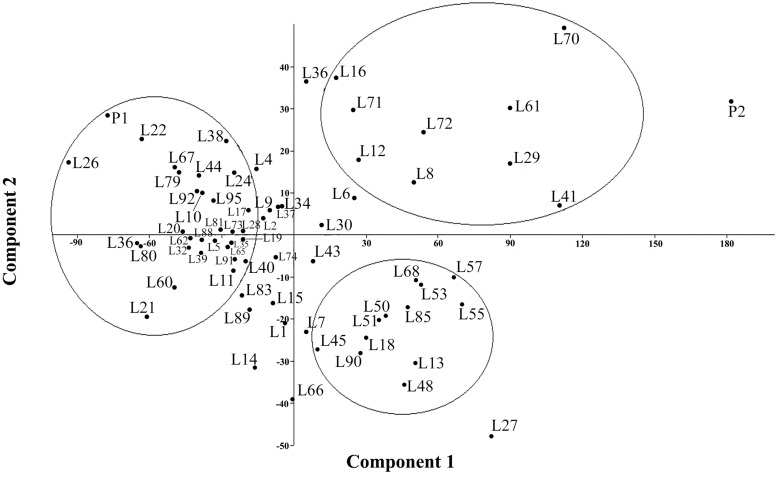
**Principal Component Analysis (PCA) plot for the variables listed in Table 5**.

The 72 studied lines represented a wide range of morphological variability that was reflected in seed and panicle coloration, plant and panicle size and shape, seed diameter, grain yield, maturity, and resistance to downy mildew (**Figure 1**). The population exhibited segregation for seed color, with 46% of the RIL’s having white seeds and 22% having red seeds – the two main coloration patterns. The remaining lines were yellow (15%), white-speckled red (3%), yellow speckled with red (7%), and light-yellow or orange (3% each).

Forty-six percent of the RIL’s in the population had green stems and light green leaves (NL-6 parental type), 43% had red stems and dark green leaves (0654-type), and the rest of the lines (8%) were heterogeneous, having green stems streaked with red and light green leaves or fully red stems and light green leaves.

With respect to panicle variation, 35% of the RIL’s had yellow panicles like P1, 25% were purple like P2, 19% had red panicles, and 3% had pink panicles. Several RIL’s exhibited panicle color segregation or heterogeneity, including yellow/red, yellow/purple, orange/ red, yellow/purple, and orange/purple. In addition to panicle color, the amaranth panicle shape (amaranthiform) was present in 60% of the RIL’s, with the remaining 39% exhibiting globular (glomerulate) panicles.

Branching pattern of the main stem was another discriminating trait. Sixty-nine percent of the RIL’s did not exhibit branching. Since branching is a wild-type trait and neither parent normally has a branched stem, the presence of significant stem branching in ~30% of the lines indicates the two parents may have complementary genes suppressing stem branching.

### Quantitative Traits

All 16 quantitative traits showed very highly significant differences among the RIL’s for practically all the studied characters, providing strong evidence for the important diversity generated within the Pop39 and highlighting the relevant discriminating power of the variables used. Seed diameter, for example, varied from 1.71 to 2.11 mm and there were highly significant differences among lines, with 28% of the RIL’s having larger seeds than the 0654 parent.

Sorting RIL’s by plant height at maturity differentiated the population into five classes or groups, producing a distribution skewed slightly toward shorter plant height. The three intermediate classes – classes 2, 3, and 4 –encompassed more than 80% of the lines (**Table 1**). At maturity, the NL-6 parent was 63.90 cm tall and thus fell into in class 1; the 0654 parent at 214.88 cm height was at the upper end of class 5. Consequently, transgressive segregants for plant height were not detected in the population.

**Table 1:** Main traits class’s interval, average, and lines’ percentage in quinoa F_2:6_ RIL Pop39.

Traits	Class	Interval	Average	Number of lines	Lines’ %
PHM (cm)	1	61–90	76.24	6	8.33
	2	90–115	105.13	23	31.94
	3	115–140	124.94	21	29.17
	4	140–170	159.31	15	20.83
	5	170–215	193.06	7	9.72
GY (g/pl)	1	0.13–4.50	2.54	15	20.83
	2	4.5–8.0	6.64	21	29.17
	3	8.0–12.0	9.62	22	30.56
	4	12.0–17.0	14.19	9	12.50
	5	17.0–23.0	20.48	5	6.94
DM (days)	1	124–131	127.15	10	13.89
	2	131–140	134.47	15	20.83
	3	140–153	147.2	24	33.33
	4	153–70	162.01	18	25.00
	5	170–192	187.31	5	6.94
RPV (scores)	1	0.5–1.2	1.0	8	11.11
	2	1.4–1.7	1.5	12	16.67
	3	1.8–2.1	2.0	13	18.06
	4	2.2–2.3	2.3	15	20.83
	5	2.4–2.5	2.5	7	9.72
	6	2.6–3.0	2.7	17	23.61

*PHM, Plant height at maturity; GY, grain yield; DM, days to maturity; YPV, resistance to peronospora variabilis. RPV scores: 1 = very resistance, 2 = resistance, 3 = slightly resistance, 4 = slightly susceptible, 5 = susceptible, 6 = very susceptible*.

Descriptive and variance analyses of biomass and its components highlighted the important diversity present among the F_2:6_ lines. The main stem diameter varied between 0.54 and 1.89 cm (**Table 2**). The NL-6 parent had a diameter of 0.71 cm and for parent 0654 it was 1.15 cm. The main root length of the whole population averaged 10.15 cm and varied among lines between 8.50 and 12.80 cm. The panicle length also presented a wide variation among lines; it was three times greater in line L70 (120.70 cm) compared to L26 (36.90 cm). The panicle width also varied, ranging from 3.61 to 17.50 cm in lines L26 and L90, respectively (**Table 2**).

**Table 2:** Biomass and its components in F_2:6_ quinoa Pop39.

Character	Mean	Max	Highest line	Min	Lowest line	*SD*
SD (cm)	0.83	1.89	L53	0.54	L26	0.42
RL (cm)	10.15	12.80	L8	8.50	L39	1.85
IL (cm)	66.85	120.70	L70	36.90	L26	26.18
IW (cm)	9.35	17.50	L90	3.61	L26	5.13
FWA (g)	51.52	196.85	0654	10.17	L21	43.21
FWR (g)	4.60	12.94	0654	0.83	L21	4.02
DWA (g)	18.14	70.79	0654	2.68	L26	16.39
DWR (g)	2.27	7.47	L90	0.47	L26	2.01
BM (g/plt)	29.11	79.39	0654	6.19	L26	21.91

*SD, main stem diameter; RL, root length; IL, inflorescence length; IW, inflorescence width; FWA, above ground fresh weight; FWR, root fresh weight; DWA, above ground dry weight; DWR, root dry weight; BM, biomass. SD, standard deviation*.

Lines could be classified into five biomass groups, with the NL-6 parent falling into class 2 (13.16–20.10 g) and 0654 grouping with class 5 (50.52–79.39 g). The aboveground and root biomasses had values for the 0654 parent of 196.85 and 12.94 g, respectively (**Table 2**). The 0654 parent also presented the highest stem dry weight (70.79 g). Total plant dry biomass varied between 6.19 and 79.39 g for lines L26 and 0654, respectively. Standard deviations were variable among characters, being very high for the fresh weight of the aboveground plant and the length of the panicle. In contrast, standard deviations were minimal for stem diameter, plant size, and root dry weight (**Table 2**).

Five seed yield classes were identified, with >80% of the lines falling into the three lowest classes and yielding less than 12 g. The 0654 parent grouped in class 1 and yielded only 2.20 g, while NL-6 yielded 12.18 g (class 4). Five of the RIL’s in class 5 produced 17–23 g of seeds per plant, indicating the potential for positive transgressive segregation for yield in Pop39. The grain yield measured per individual ranged between 0.13 and 22.42 g; the average yield of the whole F_6_ population was of 8.70 g/plant (**Table 1**). Thus, variation for seed production was highly significant in the population.

The 70 F_6_ quinoa lines grouped into five precocity classes when sorted by their number of days to maturity (**Table 1**), with a population DM average of 148 days and a standard deviation of 16.78 days. About 65% of the F_2:6_ lines required less than 5 months to mature (<150 days). The remaining 35% of lines required >150 days to mature and grouped in classes 4 and 5. The ANOVA for DM, as with the other characters, had a highly significant *p*-value. The NL-6 parent was earlier than any of the RIL’s, having reached maturity after only 124 days (**Table 1**). The 0654 parent, L48, and L90 were the latest-maturing genotypes at 192 days.

### Downy Mildew Resistance

Analysis of variance for resistance to mildew showed significant differences among lines from the second day of the inoculation (**Table 3**). *Perenospora variabilis* symptoms did not show up right after the inoculation on all the RIL’s – indicating that initial reaction to the pathogen depends on the genotype. The 70 F*6* lines were classified in six groups (**Table 1**), with the most resistant ones in class 1 (L55, L7, L27, L41, and L89) and the most susceptible in class 6 (L37, L12, L18, L92, L44, and L95). The NL-6 parent was slightly susceptible to mildew while 0654 was among the most resistant. Fifty percent of the accessions (36/72) were classified between the two parents, indicating that transgressive segregation for resistance was substantial in Pop39. The resistant 0654 parent did not show any sporulation at day 12 from the inoculation; this behavior was also the case for other lines of the three first classes. Spores appeared on the 10th day on NL-6, which was also the case in lines falling into the other three susceptible classes. The most highly susceptible lines developed their first symptoms well before 10 days after inoculation.

**Table 3:** ANOVA for resistance to mildew (*Peronospora variabilis*) infection in Pop39.

	Sum of squares	Mean squares	Mean	*SD*	F Fisher	*P*r > *F*
2 days	30.388	0.428	0.488	0.554	1.732	0.003
4 days	85.860	1.209	1.484	0.901	1.969	<0.0001
6 days	133.950	1.887	2.223	1.040	2.772	<0.0001
8 days	144.923	2.041	2.758	1.097	2.595	<0.0001
10 days	181.966	2.563	3.428	1.095	4.908	<0.0001
12 days	191.798	3.144	4.173	1.143	7.947	<0.0001

Collected data showed that the pathogen infected every single RIL; however, the most susceptible genotypes rapidly developed symptoms and allowed for sporulation, whereas resistant lines delayed the progression and spread of disease, preventing spore growth and dissemination.

### Heritability

Broad-sense heritability and variances for morphological characters are presented in **Table 4**. Phenotypic variances were greatest for PH (1154.7) and FWA (1066.97). The highest value of genotypic variance was also noted for PH (838.53); SD presented the lowest genotypic variance (0.017). Highest heritability values were observed for DM (89%) and PH (73%). Heritabilities were intermediate for DWA (55%), IL (46%), and BM (46%). Root length had the lowest heritability at 10%. Grain yield heritability was measured at 42% in this population and environment.

**Table 4:** Variance and broad-sense heritability components of 12 traits in quinoa F_2:6_ RIL Pop39.

Character	Phenotypic variance	Genotypic variance	Environmental variance	Heritability
Plant height at maturity (cm)	1154.70	838.53	316.17	0.73
Inflorescence length (cm)	588.55	271.00	317.55	0.46
Inflorescence width (cm)	19.67	7.99	11.68	0.40
Seed diameter (cm)	0.047	0.017	0.03	0.36
Root length (cm)	208.30	0.30	208.00	0.10
Above ground fresh weight (g)	1066.97	423.33	643.64	0.39
Above ground dry weight (g)	174.46	96.68	77.78	0.55
Root fresh weight (g)	7.36	2.81	4.55	0.38
Root dryweight (g)	2.28	0.96	1.32	0.42
Biomass (g/pl)	335.23	155.25	179.98	0.46
Grain yield (g/pl)	40.38	17.08	23.30	0.42
Days to maturity (days)	262.76	235.37	27.39	0.89

### Principal Component Analysis

Multivariate analysis was undertaken to highlight genotype groups with similar traits and significant correlations among characters. The principal components analysis (PCA) was computed to assess the contributions of individual variables to the global variance. Fifteen quantitative traits were used and data analysis showed that the four first components explained 96% of the variance. These 15 traits’ contributions are presented in **Table 5** for each of the first four axes. The first principal component (PC1) explained 74% of the total variance. The variables positively correlated to PC1 were PH (0.92); IL and IW (0.85 and 0.64, respectively); SD (0.69); BM (0.94); and DM (0.65). Resistance to mildew was negatively correlated to PC1 (-0.46). Grain yield was correlated to PC2 (0.6), to which DM was negatively correlated (-0.47).

**Table 5:** Variables contribution to the four principal axes in quinoa F_2:6_ RIL Pop39.

Character	Axis 1	Axis 2	Axis 3	Axis 4
Plant high at day 60	0.0650	-0.0968	0.6565	0.3918
Plant high at day 75	0.1629	-0.0308	0.8131	0.3110
Plant high at day 90	0.4522	-0.1115	0.8343	0.2193
Plant high at day maturity	0.9203	-0.3676	0.0480	0.0026
Inflorescence length (cm)	0.8503	0.1523	0.2153	-0.4385
Inflorescence width (cm)	0.6432	0.2206	0.0569	-0.1345
Seed diameter (cm)	0.6985	0.1068	0.1202	0.0360
Root length (cm)	0.3010	0.1763	0.1327	-0.0476
Above ground fresh weight (g)	0.9093	0.3786	-0.1027	0.1235
Above ground dry weight (g)	0.9591	0.0183	-0.1688	0.0238
Root fresh weight (g)	0.7962	0.2265	-0.0886	0.0139
Root dry weight (g)	0.8589	0.0792	-0.0944	-0.0632
Biomass (g/pl)	0.9433	0.2053	-0.0096	-0.0542
Grain yield (g/pl)	0.3200	0.6045	0.4417	-0.2231
Days to maturity (days)	0.6549	-0.4757	-0.4734	0.0613
Mildew reaction	-0.4647	0.2946	0.0707	-0.0330
Variance	74%	11%	9%	3%
Sum of the variance	74%	85%	93%	96%

According to the correlation matrix in **Table 6**, the most significant relationships among traits were GY with IL (0.52) and BM (0.52); DM with PHM (0.71), DWA (0.72), DWR (0.64), and BM (0.54). Susceptibility to mildew (SM) was negatively correlated to PHM (0.54) and DM (0.47).

**Table 6:** Correlation matrix among variables measured in F_2:6_ RIL quinoa Pop39.

0	PHM	IL	FWA	DWA	FWR	DWR	BM	GY	DM
PHM									
IL	0,72421								
FWA	0,69635	0,75655							
DWA	0,85625	0,76911	0,88701						
FWR	0,62341	0,69904	0,8136	0,79851					
DWR	0,72522	0,76126	0,7972	0,88355	0,93488				
BM	0,78207	0,84317	0,9138	0,95209	0,81857	0,88611			
GY	0,10011	0,52115	0,43149	0,23385	0,3125	0,29107	0,51739		
DM	0,71335	0,38958	0,46564	0,71753	0,51038	0,6402	0,53786	-0,31201	
SM	-0,53602	-0,31489	-0,32164	-0,45151	-0,40435	-0,43732	-0,38074	0,06723	-0,468

**Figure 1** presents the PCA plot of Pop39 F_2:6_ RIL’s for PC1 and PC2. On the positive side of axis 1 are grouped 15 entries – 0654 (P2), L70, L41, L61, L29, L27, L55, L57, L72, L53, L68, L13, L8, L85, L48 – that shared the characteristics of tall plant height, long and wide panicles, large stalk diameter, large biomass, and relatively long growth cycle. On the negative side of axis 1, RIL’s L60, L67, L21, L22, L80, L31, NL-6 (P1), and L26 presented lower values of the aforementioned characters. Lines on the positive side of axis 2 (L70, L16, L36, and NL-6) are characterized by higher grain yield and earlier maturity; on the negative side of axis 2 are RIL’s that matured later and had lower seed yield, notably lines L13, L14, L48, L27, and L66.

### Dendrogram

**FIGURE 2:**
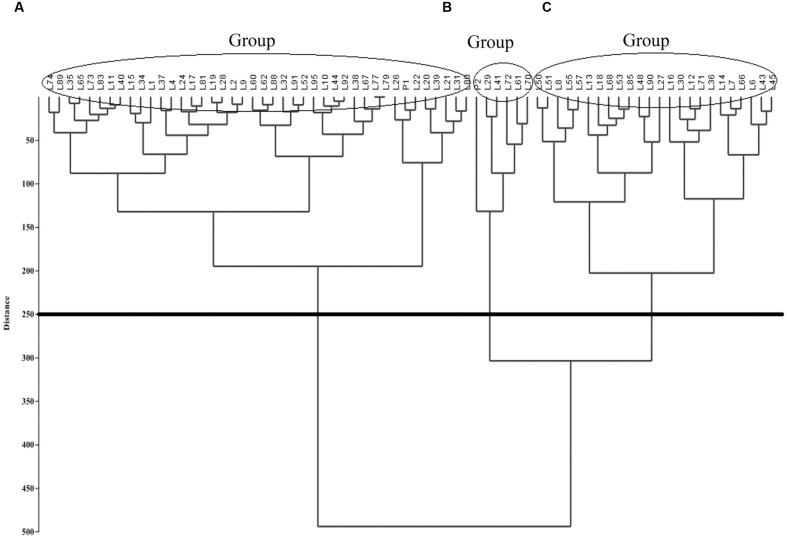
**Unweighted Pair Group Method with Arithmetic Mean (UPGMA)-based dendrogram showing three clusters (A–C) of RIL’s in quinoa Pop39**.

A dendrogram was computed based on UPGMA using PAST v. 2.16 (Hammer et al., 2001). The F2:6 quinoa RIL Pop39 separated into three clusters (**Figure 2**). Group A included short-stature lines (61–140 cm) with small panicles (36.9–69.4 cm) and low biomass (6.19–29.56 242 g). With the exception of lines L9 and L89, they were also early maturing and susceptible to *P. variabilis*. Cluster A also included the NL-6 parent (P1).

Cluster B included six lines (0654 or P2, L29, L41, L72, L61, L70), all of tall size (140–215 cm) and having both long (90.2–120.7 cm) and wide (12.63–16.14 cm) panicles. They were also of high biomass (50.52–7.39 g) and high grain yield (12–23 g). Parent 0654 was an exception because it yielded only 2.20 g/pl. However, 0654 and line L41 were very resistant to mildew, L70 and L29 had intermediate levels of resistance, while L61 was very susceptible.

Cluster C (**Figure 2**) consisted of 24 lines of somewhat large size (115–170 cm), long panicles (57.8–120.7 cm), high biomass (20.77–48.02 g), and late maturity (140–192 d). These RIL’s were mostly resistant to downy mildew, except for highly susceptible lines L30, L12, and L18.

## Discussion

The presented cross operates between two contrasting lines, NL-6 adapted to European lowlands and of short size (63.90 cm), and 0654 from the Peruvian valleys and taller (214.88 cm). There is a large genetic distance between F_2:6_ accessions, and much variability within the population. Several authors reported that the valley ecotype is generally taller than highland ecotype (Gandarillas, 1979; Cáceres, 1993; Mujica and Jacobsen, 1999; Carmen, 2008).

The F_2:6_ quinoa RIL’s presented a large genetic variability, which is translated to qualitative traits of stem, leaf, inflorescence and seed color diversity, and also by inflorescence shape and size variation. Seed color varies from black, brown, red, pink, yellow, orange to white. The inflorescence presents a very wide range of colors from white, yellow, pink, red to dark red, brown, and purple. Within the F_2:6_ accessions, the leaf color was light green in 47% RIL’s or dark green in 45%; the stems were red (43%) or green (46%) and the inflorescence were mainly yellow (35%) or red (25%), but also orange, pink, purple.

Several authors have investigated the genetic control of a number of qualitative characters. A single gene with several alleles controls leaf color, red color is dominant over purple, dominating over green. The red color is dominant at leaves axils (Tapia et al., 1979; Izquierdo Fernández et al., 2001; Carmen, 2008). Concerning the panicle shape, a 3:1 segregation of glomerulate upon amaranth forms was reported (Carmen et al., 2008). Male sterility identified in quinoa is nucleocytoplasmic, with three fertile plants for one sterile (Izquierdo Fernández et al., 2001; Carmen et al., 2008).

There is a wide range of seed colors being white, yellow, pink, dark red, brown, and purple (Cusack, 1984; Risi and Galwey, 1989). The genetic determination of this trait requires studying several cross descendants’ between homozygote lines with contrasted seed colors. The tetraploid status of the species has to be taken into account while analyzing the offspring.

The UPGMA clustering and PCA showed that the highland-phenotype, relatively unadapted lines in Rabat’s coastal climate formed a distinct group far apart from the other clusters. Highland-phenotype accessions– those most like the Peruvian 0654 parent – are of tall stature and have a more indeterminate growth pattern when cultivated in the mild and humid coastal environment. Moreover, since 0654 is susceptible to severe decreases in seed yield when exposed to elevated temperatures during flowering (personal observations), low-yielding lines may have inherited this characteristic from 0654.

Maughan et al. (2012) reported that single-nucleotide polymorphism (SNP) markers identified in this same population (Pop39) displayed 95% normal (non-distorted) segregation, with most of the skewed markers favoring the NL-6 alleles. They further speculated that this pattern of segregation distortion favoring SNP alleles from the NL-6 parent might be due to heat-induced sterility coming from the 0654 parent, even though the population was advanced to homozygosity each generation under relatively mild (25°C) temperatures in the greenhouse. Consequently, it would be interesting in a future study to address the question of whether or not these NL-6 skewed regions carry alleles for heat tolerance.

This highland RIL’s are, however, highly resistant to downy mildew; they develop small lesions and have a long latent period when inoculated with oospores of *P. variabilis* under controlled conditions. The pathogen affected most genotypes at different intensities. 0654 parent was ranked among the most resistant F_2:6_ RIL’s group, probably carrying the resistance factor. This assumption is in agreement with Kitz (2008) from the same population. The highest resistance was that of RIL L55, which confirms our earlier greenhouse observations (unpublished).

According to Alfano and Collmer (1997), Campbell et al. (1999), and Eulgem et al. (2004), the valley ecotype is usually more resistant to downy mildew than early and short size lowland ecotype, corresponding to group A of the dendrogram. These results are in concordance with Bonifacio and Saravia (1999).

The data analyses confirmed the significant diversity of the quantitative traits. Seed yield was positively correlated to panicle length (0.52) and total biomass (0.52). Bhargava et al. (2007) state good correlation between grain yield and plant biomass while Carmen (2008) reports positive correlations with plant size, panicle width, total dry matter and crop earliness.

Mujica (1988) puts up two genetic indexes that are based on high inheritability traits that are highly correlated to grain yield; those are central glomeruli diameter (Index I1) and stem and panicle diameters (Index I2). The efficiency of the two indexes reached 144.48 and 148.32%, respectively; they represent relevant parameters for germplasm evaluation and selection studies (Mujica, 1988).

Bertero et al. (2004) reported striking disparities for quinoa character correlations between cold/highland and lowland/tropical environments. For example, they reported grain yield versus biomass correlations of 0.92 and 0.48 in cold/highland versus warm/lowland test sites, respectively. Although Rabat is on the coastal plain and at mid-latitude, its proximity to the ocean moderates the temperature; nonetheless, the observed correlation of yield versus biomass of 0.52 in this study follows the heat-stress pattern detected by Bertero et al. (2004). Biomass was still a close second behind (IL, 0.521) for magnitude of correlation with grain yield in this study.

Bertero et al. (2004) also reported a disparity for quinoa grain yield-maturity correlations, having observed values of 0.87 and 0.37 in cold/highland and warm/lowland environments, respectively. In the present study, we found that grain yield and days to maturity were negatively correlated at -0.31. This is most likely due to later-maturing lines encountering debilitating late spring-summer heat stress, especially for lines carrying alleles from the heat-susceptible parent 0654.

Bertero et al. (1999) reported strong effects on seed size and maturation from high temperatures and post-anthesis exposure to long days in Altiplano (variety ‘Kanckolla’) and Valley (variety ‘Blanca de Junin’) quinoa genotypes. Since our parental cultivar ‘0654’ is of similar intermediate-late maturity and environmental adaptation as ‘Blanca de Junin’, it is not surprising that plant height, inflorescence size, biomass, seed diameter, and maturation all contributed heavily to Axis 1 of the PCA (**Table 5**). With a February sowing date at Rabat (~34° N latitude), lines having greater allelic contributions for daylength sensitivity from ‘0654’ would be expected to have delayed maturation, higher biomass, etc., whereas daylength-neutral alleles from the coastal genetic background of ‘NL-6’ would have contributed to markedly earlier maturation and relatively reduced biomass parameters under the same lengthening-day conditions – and without transgressive segregation for maturation from ‘NL-6’ contributing to a longer growth cycle.

Clearly, salient breeding objectives in the mild coastal climate of Rabat should include higher seed yield, shorter growing cycle and downy mildew resistance – though given the correlation between more lengthy growth cycle and higher mildew resistance, and in light of Rabat’s lack of severe summer heat or winter freeze constraints, mildew resistance probably should not be sacrificed for earlier maturation. This is based on the presumption that the humid climate in Rabat is conducive to mildew spore exposure and growth year-round. Nevertheless, evaluations of larger numbers of segregating progeny from lowland × highland quinoa populations like Pop39 should facilitate uncoupling of traits like heat tolerance/early maturity + mildew susceptibility.

The extensive morphological variation present in just 70 F2:6 lines of the NL-6 × 0654 RIL population, combined with the pattern of relatively moderate linkage distortion detected in this population using molecular markers (Maughan et al., 2012), bodes well for the continued use of lowland coastal × Andean highland quinoa breeding populations. The detection of positive transgressive segregants for seed size and yield, in particular, combined with an observed heritability for yield of approximately 40%, bodes well for future selection to develop higher-yielding quinoa lines for the Moroccan coastal environment.

## Author Contributions

OB and NB designed the study. PM and EJ prepared the population. NB and OB collected the data. NB, OB, and SJ analyzed the data. OB, SJ, PM, and EJ obtained funding to support this project. NB, OB, and EJ took primary responsibility for writing the manuscript.

## Conflict of Interest Statement

The authors declare that the research was conducted in the absence of any commercial or financial relationships that could be construed as a potential conflict of interest.
